# Integrated Network Pharmacology and Molecular Docking Uncover Multi-Target Actions of *Cladophora glomerata*–Derived Compounds Against Chronic Obstructive Pulmonary Disease

**DOI:** 10.3390/ijms27041619

**Published:** 2026-02-07

**Authors:** Anis Ahamed Nazeer, Ahmed E. Al-Sabri, Salah N. Sorrori, Ibrahim A. Arif

**Affiliations:** 1 Department of Botany and Microbiology, College of Science, King Saud University, P.O. Box 2455, Riyadh 11451, Saudi Arabiaiaarif@ksu.edu.sa (I.A.A.); 2 Plant Production Department, College of Food and Agriculture Sciences, King Saud University, Riyadh 13362, Saudi Arabia

**Keywords:** COPD, *Cladophora glomerata*, network pharmacology, molecular docking, *EGFR*, *TP53*

## Abstract

Chronic Obstructive Pulmonary Disease (COPD) is a complex inflammatory lung condition characterized by oxidative stress, changes in airway structure, and gradually worsening airflow blockage. Existing treatments offer only symptomatic management, emphasizing the need for multi-target therapeutic interventions. This study employed a combined approach of network pharmacology and molecular docking to investigate the therapeutic effects of bioactive compounds derived from *Cladophora glomerata* on COPD. Disease-associated genes were collected from GeneCards, Online Mendelian Inheritance in Man (OMIM), and National Center for Biotechnology Information (NCBI), while compounds from *C. glomerata* and their predicted molecular targets were obtained from SwissTargetPrediction. A cross-comparison of targets related to compounds and diseases revealed nine common genes, among which three central genes *TP53, CASP8*, and *EGFR* were identified using protein–protein interaction (PPI) network analysis. Analysis of gene–disease interactions highlighted Tumor Protein p53 (*TP53*) and Epidermal Growth Factor Receptor (*EGFR*) as major regulatory targets. GeneMANIA-based functional and co-expression analysis revealed predominant physical interactions (77.64%) and co-expression relationships (8.01%), highlighting strong functional connectivity among the identified genes. Molecular docking further confirmed that *C. glomerata* derived compounds, particularly Quinoline, 1,2,3,4-tetrahydro-1-((2-phenylcyclopropyl)sulfonyl)-, trans- (**Pubchem ID: 91709903**) (−7.5 kcal/mol) and1,2,4-Oxadiazole, 3-(1,3-benzodioxol-5-yl)-5-[(4-iodo-1H-pyrazol-1-yl)methyl]- (**Pubchem ID: 5301194**) (−7.3 kcal/mol), exhibit favorable predicted binding affinities toward EGFR and TP53 in molecular docking analysis. Overall, these insights suggest that *Cladophora glomerata* compounds may modulate key COPD-related pathways through multi-target interactions, providing a scientific basis for future experimental studies and the development of marine-derived therapeutic agents for COPD management.

## 1. Introduction

Globally, COPD impacts over 384 million individuals, with evidence indicating that more than half of them may not receive appropriate treatment. The World Health Organization estimates that COPD costs more than $100 billion annually and is the third most common cause of death worldwide [1]. COPD arises from multiple contributing factors, including pulmonary-driven systemic inflammation, age-associated comorbidities, cigarette smoke and other inhaled toxic agents, as well as reduced physical activity, inadequate nutrition, and hypoxemia [2]. A continuous inflammatory reaction mainly affecting the lung tissue and nearby airways is a key factor in the onset and development of the disease, eventually causing permanent airflow blockage and chronic respiratory symptoms. This chronic inflammation is characterized by elevated numbers of neutrophils, lymphocytes, and macrophages within the lung parenchyma, peripheral airways, and pulmonary vasculature. In some people, there may be elevated amounts of eosinophils, T helper 2 (Th2) cells, and type 2 innate lymphoid cells (ILC2s). These immune cells, together with lung structural cells, produce various inflammatory substances that contribute to tissue injury and changes in the airway structure. Oxidative stress further amplifies inflammation associated with both smoking exposure and COPD pathology. Additionally, COPD patients often exhibit systemic inflammatory responses, which can exacerbate metabolic dysfunction, cardiovascular complications, and other comorbid conditions [3]. The critical need for innovative therapeutic approaches that not only address the complicated pathophysiology of COPD but also offer more accessible, multi-targeted interventions is highlighted by these epidemiological and diagnostic difficulties [4].

Green algae seaweeds called *Cladophora glomerata* are frequently seen in Polish bays. One The majority of these species have been found in the Atlantic, Pacific, and Indian oceans. In Rameshwaram, Gulf of Mannar, 28 species of the Chlorophyceae family have been recognized. The majority of the planet is submerged in water, which is home to a variety of aquatic plants with medicinal properties [5]. This species contains high levels of proteins, fats, carbohydrates, vitamins, and minerals. Moreover, extracts from *Cladophora glomerata* (CGE) have demonstrated significant anti-inflammatory, blood pressure-lowering, and antioxidant effects in both laboratory and live organism studies. Previous research indicates that CGE influences protein kinases C and ζ, thereby safeguarding renal organic anion transport and exerting antidiabetic effects [6]. This moss is rich in diverse phytochemicals, including flavonoids, alkaloids, phenols, tannins, fatty acids, sterols, and terpenoids. The proliferation of this annual filamentous macroalga is largely driven by elevated nutrient levels particularly nitrogen and phosphorus in aquatic environments [7]. Numerous investigations have found new phytochemicals in this marine plant that have various pharmacological properties. For many years, algae have been considered a possible source of nutrition and pharmaceuticals [5].

In the field of drug development, network pharmacology (NP) is a rising star because it combines information science with systematic medicine. A “protein-compound/disease-gene” network is introduced using an integrated in silico method to uncover the reasons behind the complementary therapeutic effects of conventional medications [8]. Network pharmacology views disease processes as linked networks that can be better treated using several complementary drugs. In recent years, this approach has become increasingly valuable for elucidating the pharmacological actions of herbal medicines. The term “network pharmacology,” first introduced by Hopkins in 2007, is based on the concept that potent therapeutic effects often arise from compounds acting on numerous targets rather than a single molecular site [9]. Molecular docking is a computational technique employed to investigate how protein receptors interact and recognize small-molecule ligands, enabling the prediction of their binding orientation and affinity strength [10]. Molecular docking is an essential element of rational drug design. Owing to its biological and pharmacological importance, substantial efforts have been devoted to improving docking algorithms to achieve more accurate predictive performance [11]. A common method for verifying possible interactions between active substances and target genes found by network pharmacology is molecular docking studies [12].

This research utilized network pharmacology and molecular docking methods to assess the potential therapeutic relevance of compounds derived from Cladophora glomerata against COPD, providing a scientific basis for its possible clinical application while offering deeper insight into its mechanistic roles; however, as this study is entirely computational, the predicted interactions require validation through in vitro and in vivo experimental studies.

## 2. Results

### 2.1. Retrieval of Copd-Associated Target Genes

**Figure 1:**
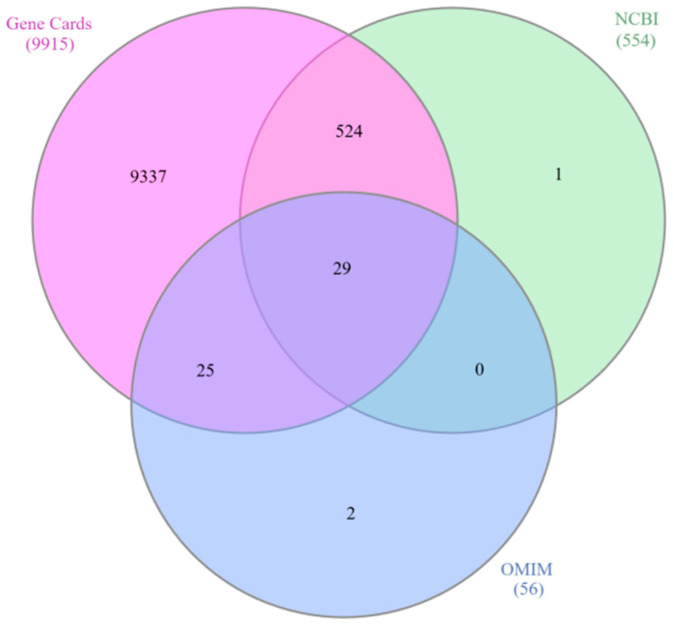
Venn Diagram of COPD-associated genes retrieved from GeneCards, OMIM and NCBI Databases.

From the GeneCards database, 9915 COPD-associated genes were identified, along with 554 genes from NCBI and 56 from OMIM (Sheet S1). Interactive Venn analysis was then used to determine the shared genes across these datasets, yielding 29 common COPD-related targets (Figure 1) (Sheet S2).

### 2.2. Identification of Bioactive Compounds Associated with Cladophora glomerata

Bioactive compounds derived from *Cladophora glomerata* were collected through an extensive literature survey. Only compounds with previously reported biological or pharmacological activity and available structural information in PubChem were included for further analysis, and their related 3D structures and SMILES representations were retrieved from the PubChem database for further computational studies (Table S1).

### 2.3. Retrieval of Compound-Related Targets

The molecular targets associated with the identified compounds were predicted using the SwissTargetPrediction database, and all predicted targets were included to ensure comprehensive coverage of potential compound–target interactions, yielding 1638 potential targets (Sheet S3). After eliminating duplicates, 617 unique compound-related targets (Sheet S4) were obtained for further analyses. This screening step resulted in a quantitative reduction in targets, improving the reliability of compound–disease target overlap.

### 2.4. Integration of Disease-Related and Compound-Related Targets

**Figure 2:**
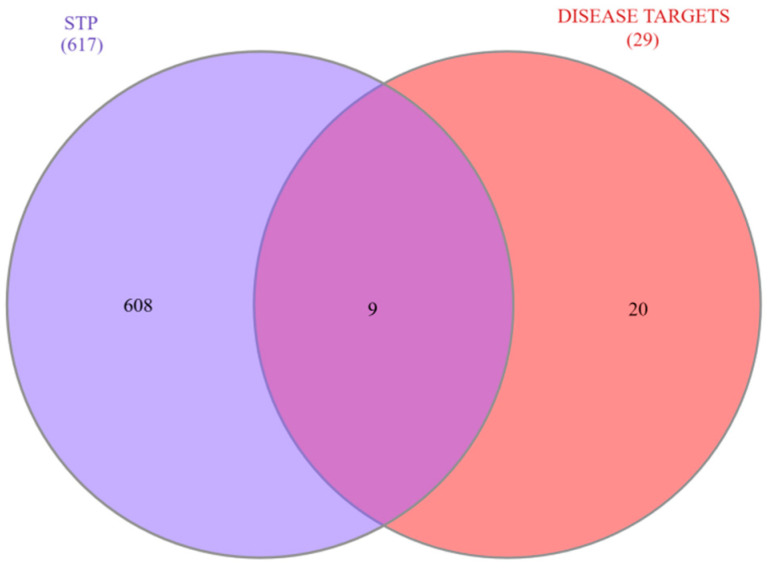
A Venn diagram was created to show the common genes between targets linked to COPD and those related to compounds from *Cladophora glomerata* as predicted by SwissTargetPrediction.

The 29 COPD-associated genes and 617 unique compound-related targets were integrated using Interactive Venn analysis to determine the overlapping targets. The analysis identified 9 common genes, representing the potential therapeutic targets of *Cladophora glomerata* compounds against COPD (Figure 2) (Sheet S5).

### 2.5. Construction of the Protein–Protein Interaction (Ppi) Network

**Figure 3:**
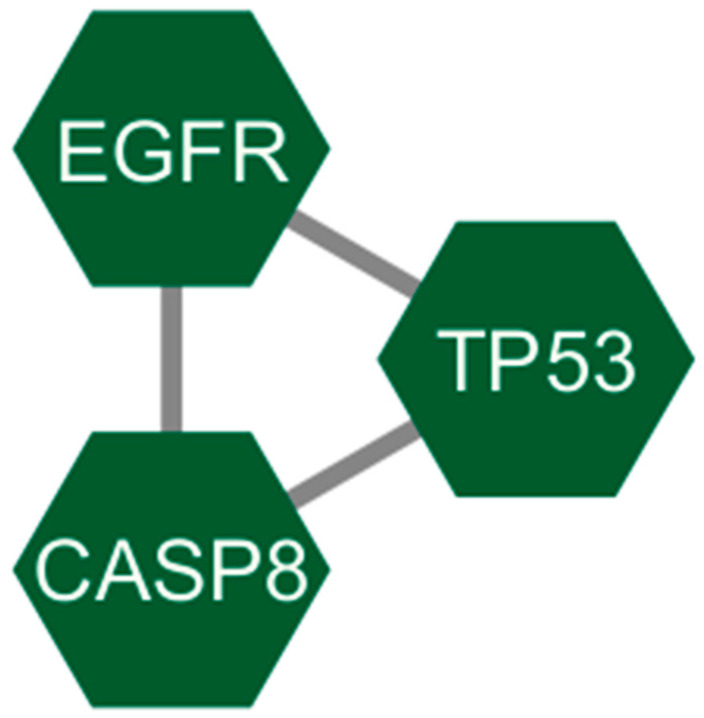
Protein–protein interaction of overlapping genes between COPD-associated genes and targets related to compounds from *Cladophora glomerata*.

The 9 common targets were examined using the STRING database to build the protein–protein interaction (PPI) network (Sheet S6). The analysis identified 3 genes TP53, CASP8 and EGFR suggesting their prominent regulatory roles in COPD-related molecular mechanisms (Figure 3).

### 2.6. Gene–Disease Interaction Network

**Figure 4:**
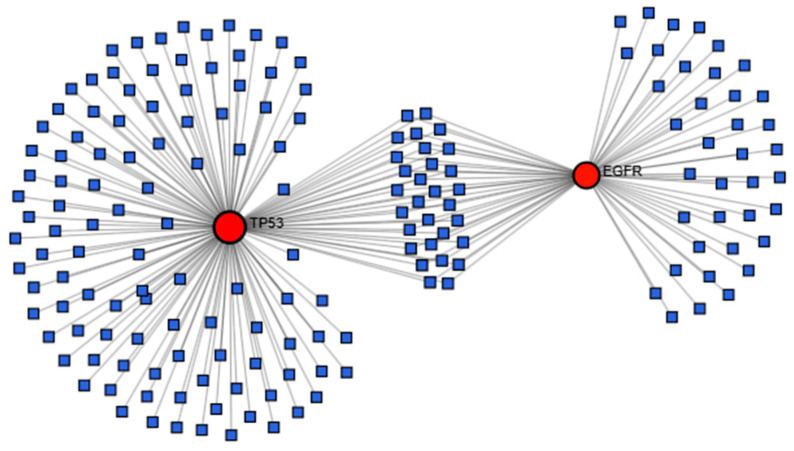
Disease and genes Interaction of overlapping genes between COPD-associated genes and Cladophora Glomerata compound related target.

**Figure 5:**
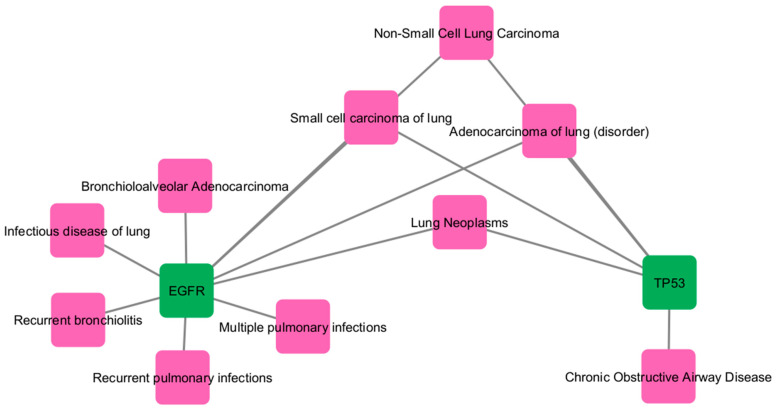
COPD related Disease and genes Interaction of overlapping genes between COPD-associated genes and Cladophora Glomerata compound related target visualized using Cytoscape. Green Circle rectangle node represent disease related genes and Violet Circle rectangle node represent COPD related disease.

The 6 hub genes were further analyzed using NetworkAnalyst to explore their disease associations. The gene–disease interaction network identified TP53 and EGFR as the most significant targets linked to COPD (Figure 4). The results were visualized in Cytoscape v.3.10.3, and these two genes were chosen for further molecular docking analysis (Figure 5) (Sheet S7).

### 2.7. Physical Interaction and Co-Expression Analysis of Key Target Genes

**Figure 6:**
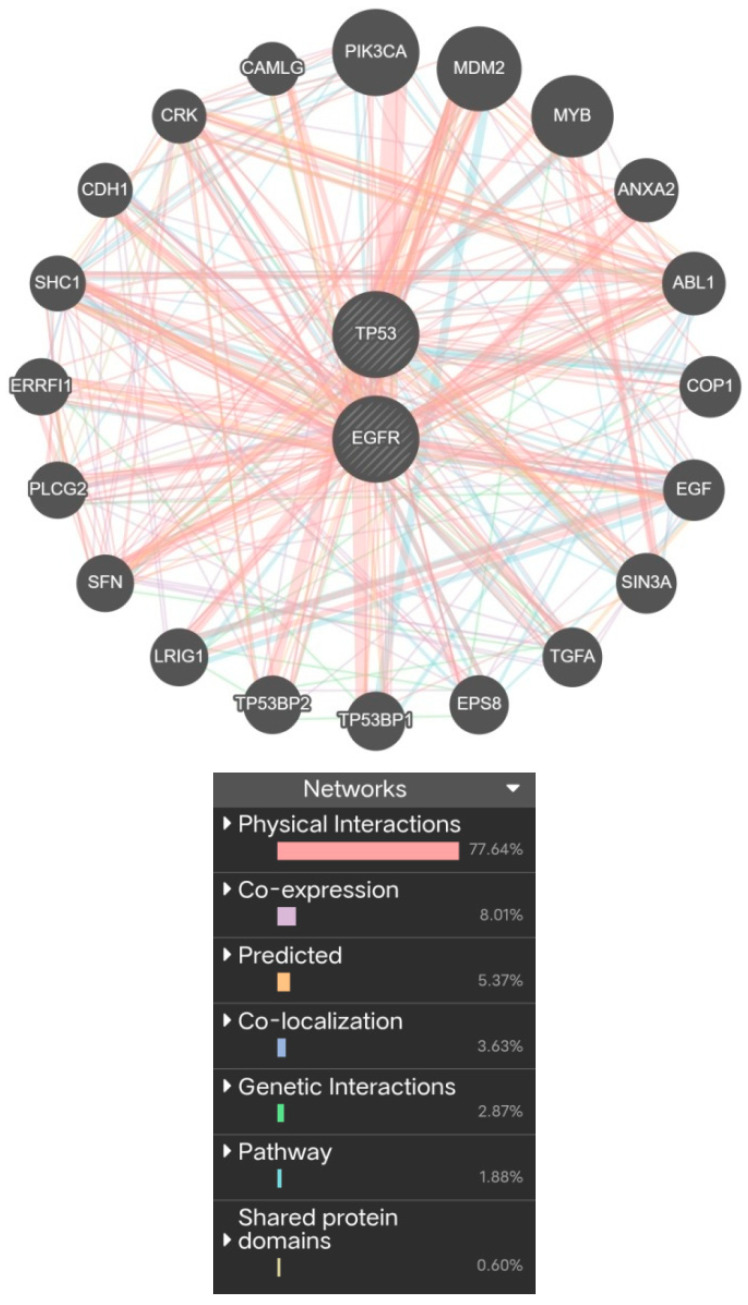
Physical Interaction and Co-Expression Interaction of Disease associated genes.

The *TP53* and *EGFR* genes were further analyzed using GeneMANIA to understand their functional associations (Sheet S8). The constructed network revealed that *TP53* and *EGFR* are predominantly connected through physical interactions, with additional contributions from co-expression and other interaction types (Figure 6). These results indicate that *TP53* and *EGFR* are primarily linked through physical and co-expression relationships, highlighting their strong synergistic involvement in COPD-related signaling pathways.

### 2.8. Protein Retrieval and Preparation

The three-dimensional structures of *EGFR* (PDB ID: 2RGP) and *TP53* (PDB ID: 2OCJ) were obtained from the Protein Data Bank and analyzed using BIOVIA Discovery Studio. Binding sites were identified using DeepSite, followed by docking of compounds derived from *Cladophora glomerata* with both proteins.

### 2.9. Molecular Docking Analysis

**Figure 7:**
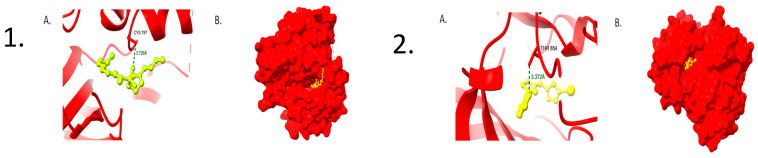
(**1**). (**A**) shows the 3D molecular interaction of *EGFR* with the Quinoline derivative (PubChem ID: **91709903**), and (**B**) shows the corresponding surface representation. (**2**). (**A**) shows the 3D molecular interaction of *TP53* with the Oxadiazole derivative (PubChem ID: **5301194**), and (**B**) shows the corresponding surface representation.

**Figure 8:**
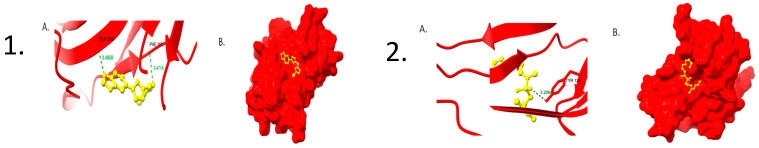
(**1**). (**A**) shows the 3D molecular interaction of *TP53* with the Oxadiazole derivative (PubChem ID: **5301194**), and (**B**) shows the corresponding surface representation. (**2**) (**A**) shows the 3D molecular interaction of *EGFR* with the Quinoline derivative (PubChem ID: **91709903**), and (**B**) shows the corresponding surface representation.

Network construction analysis highlighted *EGFR* and *TP53* as major hub proteins, underscoring their pivotal regulatory functions in molecular interactions related to COPD. To evaluate their potential as therapeutic targets, molecular docking was conducted using compounds derived from *Cladophora glomerata* against EGFR (Table S2) and *TP53* (Table S3). Docking outcomes revealed that *EGFR* displayed the strongest binding affinities with Compound **91709903** (−7.5 kcal/mol) and **5301194** (−7.3 kcal/mol). The former formed a hydrogen bond with CYS797 (3.72 Å) (Figure 7(1)) and was positioned deeply within the binding pocket, while the latter established a hydrogen bond with THR854 (3.37 Å) (Figure 7(2)), contributing to complex stability. For *TP53*, **5301194** demonstrated a binding affinity of −6.2 kcal/mol, forming hydrogen bonds with SER269 (3.48Å) and PHE113 (3.47 Å) (Figure 8(1)). Meanwhile, Compound **91709903** showed a binding affinity of −5.8 kcal/mol and engaged in hydrogen bonding with TYR126 (3.21 Å) within the active site (Figure 8(2)). Overall, the integrated network and docking analyses demonstrate that *Cladophora glomerata* compounds exhibit strong and favorable interactions with the hub proteins *EGFR* and *TP53*, highlighting their promise as potential therapeutic agents for COPD management.

## 3. Discussion

Numerous genes and pathways are involved in the pathophysiology of COPD, a complex illness involving oxidative stress, inflammatory signaling, and airway remodeling [13]. Recently, new methods for methodically identifying phytochemical target interactions related to chronic diseases have been made possible via network pharmacology and molecular docking methodologies [14]. This integrative computational framework has been widely applied to elucidate multi-target mechanisms and key regulatory nodes in disease-associated networks, as demonstrated in prior systems biology and machine-learning-assisted network studies [15].

This research utilizes network pharmacology and molecular docking techniques to explore the possible therapeutic effects of compounds derived from *Cladophora glomerata* in treating chronic obstructive pulmonary disease (COPD).By combining targets from GeneCards, NCBI, and OMIM, 29 overlapping COPD-related genes were identified, while 617 unique targets were obtained for *C. glomerata*-derived compounds through SwissTargetPrediction. The integration of these datasets revealed 9 overlapping targets, among which three genes (*TP53*, *CASP8*, and *EGFR*) were identified from the PPI network, indicating their central regulatory roles in COPD pathogenesis. Gene–disease interaction analysis identified *EGFR* and *TP53* as prominent targets. *EGFR* plays a central regulatory role in COPD pathogenesis by directly modulating airway epithelial function. Ligand-mediated activation of *EGFR* in airway epithelial cells triggers receptor autophosphorylation, leading to activation of downstream signaling pathways such as MAPK/ERK and NF-κB. These pathways induce transcriptional upregulation of mucin genes, particularly MUC5AC, resulting in goblet cell hyperplasia, mucus hypersecretion, and airway obstruction. Concurrently, *EGFR* signaling promotes the release of pro-inflammatory cytokines and chemokines, sustaining chronic airway inflammation. Persistent *EGFR* activation further contributes to epithelial remodeling and impaired barrier function, thereby exacerbating COPD progression [16,17,18]. *TP53*, on the other hand, regulates oxidative stress responses and apoptosis, contributing to lung tissue repair and cellular homeostasis in COPD [19]. In airway epithelial cells exposed to cigarette smoke-induced oxidative damage, *TP53* activation promotes cell-cycle arrest and apoptotic clearance of severely damaged cells, thereby preventing the accumulation of dysfunctional epithelial cells. Dysregulation of *TP53* signaling can impair DNA repair capacity and apoptotic responses, leading to persistent epithelial injury, chronic inflammation, and progressive lung tissue damage, which collectively contribute to COPD pathogenesis [20]. The GeneMANIA analysis also showed that these targets are mainly linked by physical interactions (77.64%) and co-expression (8.01%), highlighting a significant biological interconnection. Analogous computational pipelines have been employed in research on Salvia miltiorrhiza [21] and Trichosantheskirilowii [22] Where Network analysis highlighted EGFR and TP53 as key therapeutic nodes.

Docking analysis suggested that the bioactive compounds from *Cladophora glomerata* exhibit favorable predicted binding affinities toward the key COPD-related targets *EGFR* and *TP53*, indicating their potential relevance based on computational analysis. For EGFR, the Quinoline derivative **(PubChem ID: 91709903)** exhibited the highest affinity (−7.5 kcal/mol), followed closely by **5301194** (−7.3 kcal/mol), demonstrating stable interactions within the receptor’s active site. The docking affinity observed in this study is comparable to previously reported COPD-related analyses, where Tanshinone IIA demonstrated high binding affinity toward *EGFR*, thereby reinforcing the potential relevance of the identified interaction [23]. Likewise, for *TP53*, the 5301194 compound showed a binding affinity of −6.2 kcal/mol, while the **91709903** derivative displayed −5.8 kcal/mol. The binding affinity obtained in this study is comparable to earlier COPD-related reports in which hesperetin exhibited strong interaction with *TP53*, supporting the relevance of the predicted association [24]. The molecular docking workflow applied in this study, including ligand optimization, charge assignment, and receptor preparation, follows established and validated protocols reported in prior natural-product-based docking investigations [25]. Together, the network and docking analyses support the relevance of *EGFR* and *TP53* in COPD-associated molecular pathways.

This study highlights the value of integrated computational tools including InteractiVenn for identifying overlapping targets, SwissTargetPrediction for target prediction, and Cytoscape/NetworkAnalyst together with GeneMANIA for network construction in uncovering the multi-target therapeutic potential of *Cladophora glomerata* compounds against COPD. Among the evaluated compounds, **91709903** emerged as the most promising candidate, exhibiting strong predicted interactions with both *EGFR* and *TP53*, suggesting its role as a promising modulator of COPD-associated molecular pathways. These findings highlight the usefulness of network-based pharmacology in understanding the intricate interactions of natural substances. Nevertheless, additional experimental studies conducted in both laboratory and living organisms are necessary to verify these computational results and to confirm the therapeutic potential of compounds derived from *C. glomerata* as modulators of *EGFR* and *TP53* in the treatment of COPD.

## 4. Materials and Methods

### 4.1. Identification of Copd-Related Target Genes

Potential target genes associated with Chronic Obstructive Pulmonary Disease (COPD) were retrieved from GeneCards [26], OMIM (Online Mendelian Inheritance in Man) [27], and NCBI Gene databases [28]. The gene lists obtained from these databases were compared, and common overlapping genes were identified using the Interactive Venn tool [29]. These shared genes were identified as potential COPD-related targets and were selected for subsequent analyses.

### 4.2. Retrieval of Bioactive Compounds from Cladophora glomerata

Bioactive compounds reported from *Cladophora glomerata* were collected through an extensive literature survey of scientific publications and phytochemical reports [30,31]. The SMILES notation and 3D structures (SDF format) of the identified compounds were retrieved from the PubChem database [32]. Only compounds with known biological or pharmacological activity were selected for further study.

### 4.3. Prediction of Compound-Related Target Genes

The retrieved *Cladophora glomerata* compounds were subjected to target prediction using the SwissTargetPrediction (STP) database [33]. The SMILES structures obtained from PubChem were used as input, and predicted human protein targets were retrieved. The resulting compound-related targets were curated to remove duplicates and considered for integration with COPD-related genes.

### 4.4. Integration of Copd- and Compound-Related Targets

The overlapping targets between the predicted compound-related targets (from STP) and the COPD-associated genes (from GeneCards, OMIM, and NCBI) were identified using the Interactive Venn tool [29]. These overlapping targets represent the potential therapeutic sites through which *Cladophora glomerata* may act against COPD, and they were subsequently utilized for network construction and molecular docking analyses.

### 4.5. Protein–Protein Interaction (Ppi) Network Construction

Protein–protein interactions was constructed for the overlapping targets using the STRING (Search Tool for the Retrieval of Interacting Genes/Proteins) [34]. Interactions with a confidence score ≥0.7 were included. The interaction network was visualized and analyzed using Cytoscape software v3.10.3 [35] to identify the key interacting nodes.

### 4.6. Gene–Disease Interaction Network Construction

A Gene–Disease Interaction Network was generated using the NetworkAnalyst platform [36] to investigate disease associations and functional linkages among the identified targets. The resulting network data were exported and visualized using Cytoscape v3.10.3 [35] to highlight key gene–disease relationships and biological relevance in COPD pathology.

### 4.7. Physical Interaction and Co-Expression Network Analysis

The GeneMANIA database [37] was used to construct a physical interaction and co-expression network for the genes identified in NetworkAnalyst. This analysis provided insights into the functional associations, shared pathways, and biological processes among the selected targets. The network incorporated different types of interactions, including physical binding, co-expression, and genetic correlation, represented through distinct edges in the visualization.

### 4.8. Protein Retrieval and Molecular Docking Analysis

The three-dimensional structures of Epidermal Growth Factor Receptor (*EGFR*, PDB ID: 2RGP) and Tumor Protein p53 (*TP53*, PDB ID: 2OCJ) were obtained from the Protein Data Bank [38]. Protein preparation was carried out using BIOVIA Discovery Studioby removing water molecules and co-crystallized ligands, adding polar hydrogens, and performing energy minimization. Partial charges were assigned using Gasteiger charges, and proteins were saved in PDBQT format. Ligand geometries were optimized prior to docking to minimize structural bias and improve binding accuracy.

Binding sites were predicted using DeepSite [39]. Ligands from Cladophora glomerata were retrieved from PubChem, optimized, and converted to PDBQT format. Molecular docking was performed using AutoDock Vina v1.1.2 [40], applying default parameters with an exhaustiveness value of 8 and 10 binding modes per ligand. Docking results were ranked based on binding affinity (kcal/mol) and visualized using UCSF ChimeraX v1.10.1 [41].

## 5. Conclusions

This research emphasizes the successful application of network pharmacology, GeneMANIA network analysis, and molecular docking to reveal the multi-target treatment capabilities of compounds from *Cladophora glomerata* for COPD. By integrating compound-related and disease-associated targets, *EGFR* and *TP53* were identified as central hub proteins implicated in COPD pathogenesis, supported by strong physical and co-expression interactions within the network. Docking results demonstrated that Quinoline derivatives and Oxadiazole derivatives bind favorably to these targets, indicating their potential to modulate oxidative stress, inflammatory pathways, and airway remodeling mechanisms involved in COPD. This integrated computational strategy underscores the value of systems-level methods in revealing therapeutic mechanisms of marine-derived natural products. However, experimental confirmation using both in vitro and in vivo studies is essential to verify these results. Future validation studies may include in vitro experiments using airway epithelial or macrophage cell models to evaluate inflammatory cytokine production, oxidative stress markers, and *EGFR/TP53* signaling, along with in vivo COPD models to further confirm the therapeutic relevance of *Cladophora glomerata* compounds.

## Data Availability

The original contributions presented in this study are included in the article/Supplementary Material. Further inquiries can be directed to the corresponding author.

## Supplementary Materials

The following supporting information can be downloaded at: https://www.mdpi.com/article/10.3390/ijms27041619/s1.

## Abbreviations

The following abbreviations are used in this manuscript:

COPD	Chronic Obstructive Pulmonary Disease
*C. glomerata*	*Cladophora glomerata*
EGFR	Epidermal Growth Factor Receptor
TP53	Tumor Protein p53
MMP1	Matrix Metalloproteinase 1
MMP12	Matrix Metalloproteinase 12
HMOX1	Heme Oxygenase 1
CASP8	Caspase 8
IL6	Interleukin 6
TNF	Tumor Necrosis Factor
NFE2L2	Nuclear Factor Erythroid 2–Like 2 (NRF2)
