# Supplementary material for: Integrated Network Pharmacology and Molecular Docking Uncover Multi-Target Actions of Cladophora glomerata–Derived Compounds Against Chronic Obstructive Pulmonary Disease
Source: Int J Mol Sci. 2026 Feb 7;27(4):1619. doi: 10.3390/ijms27041619 (PMC12940951; doi:10.3390/ijms27041619)
Supplement: Supplementary file 1 [file ijms-27-01619-s001.zip › Supplementary Files/Table_S1.pdf]

Table S1: Cladophora glomerata Derived compound with their Pubchem ID, Compound Name and SMILES

S.No	Pubchem ID	Compound Name	SMILES	Citation
1	3039998	1,3-Propanediol,2-(hydroxymethyl)-2-nitro-	C1C2(COP(=O)(O1)OC2)[N+](=O)[O-]	Shah et al., 2022
2	3893	Dodecanoic Acid	CCCCCCCCCCCC(=O)O	Shah et al., 2022
3	11005	Tetradecanoic acid	CCCCCCCCCCCCCC(=O)O	Shah et al., 2022
4	69425	1-Octadecyne	CCCCCCCCCCCCCCCCC#C	Shah et al., 2022
5	95495	2-Undecanone, 6,10-dimethyl	CC(C)CCCC(C)CCCC(=O)C	Shah et al., 2022
6	5366244	3,7,11,15-Tetramethyl-2-hexadecen-1-ol	CC(C)CCCC(C)CCCC(C)CCC/C(=C/CO)/C	Shah et al., 2022
7	643801	9-Hexadecenoic acid, methyl ester,(Z)-	CCCCCC/C=C\CCCCCCCC(=O)OC	Shah et al., 2022
8	5362679	Hexadecenoic acid, methyl ester	CCCCCCCCCCCCC/C=C/C(=O)OC	Shah et al., 2022
9	5362676	Z,Z,Z-1,4,6,9-Nonadecatetraene	CCCCCCCCC/C=C\C/C=C\C=C/CC=C	Shah et al., 2022
10	5362793	9,12- Octadecadienoic acid, methyl ester(E,E)-	CCCCC/C=C/C/C=C/CCCCCCCC(=O)OC	Shah et al., 2022
11	5280435	Phytol	C[C@@H](CCC[C@@H](C)CCC/C(=C/CO)/C)CCCC(C)C	Shah et al., 2022
12	5838	Cyclobarbital	CCC1(C(=O)NC(=O)NC1=O)C2=CCCCC2	Nachammai et al., 2025
13	610088	N-Methyl-1-adamantaneacetamide	CNC(=O)CC12CC3CC(C1)CC(C3)C2	Nachammai et al., 2025
14	5301194	1,2,4-Oxadiazole, 3-(1,3-benzodioxol-5-yl)-5-[4-iodo-1H-pyrazol-1-yl)methyl]-	C1OC2=C(O1)C=C(C=C2)C3=NOC(=N3)CN4C=C(C=N4)I	Nachammai et al., 2025
15	91709903	Quinoline, 1,2,3,4-tetrahydro 1-((2	C1CC2=CC=CC=C2N(C1)S(=O)(=O)[C@H]3C[C@@H]3 C4=CC=CC=C4	Nachammai et al., 2025

		phenylcyclopropyl)sulfonyl-,trans-		
16	279315	1H-Pyrrolo[3,4-c]pyridine-1,3,4(2H,5H)-trione, 6-methyl-	CC1=CC2=C(C(=O)N1)C(=O)NC2=O	Nachammai et al., 2025
17	6427456	Bicyclo[3.1.1]heptane, 2,6,6-trimethyl, 2,3-bis-(methylthio)	C[C@@]1([C@@H](CC2CC1C2(C)C)SC)SC	Nachammai et al., 2025
