# Supplementary material for: Integrated Network Pharmacology and Molecular Docking Uncover Multi-Target Actions of Cladophora glomerata–Derived Compounds Against Chronic Obstructive Pulmonary Disease
Source: Int J Mol Sci. 2026 Feb 7;27(4):1619. doi: 10.3390/ijms27041619 (PMC12940951; doi:10.3390/ijms27041619)
Supplement: Supplementary file 1 [file ijms-27-01619-s001.zip › Supplementary Files/Table_S2.pdf]

Table S2. Binding Affinity of Cladophora Glomerata derived compounds with target protein EGFR

S.No	Compound Name	Binding Affinity (kcal/mol)
1	Quinoline, 1,2,3,4-tetrahydro 1-((2 phenylcyclopropyl)sulfonyl-,trans-	-7.5
2	1,2,4-Oxadiazole, 3-(1,3-benzodioxol-5-yl)-5-[4-iodo-1H-pyrazol-1-yl)methyl]-	-7.3
3	Phytol	-7
4	3,7,11,15-Tetramethyl-2-hexadecen-1-ol	-7
5	Z,Z,Z-1,4,6,9-Nonadecatetraene	-6.4
6	1H-Pyrrolo[3,4-c]pyridine- 1,3,4(2H,5H)-trione, 6-methyl-	-6.3
7	N-Methyl-1-adamantaneacetamide	-6.2
8	Hexadecenoic acid, methyl ester	-6.1
9	9-Hexadecenoic acid, methyl ester,(Z)-	-6.1
10	9,12- Octadecadienoic acid, methyl ester(E,E)-	-6
11	Tetradecanoic acid	-6
12	Bicyclo[3.1.1]heptane, 2,6,6-trimethyl, 2,3-bis-(methylthio)	-5.9
13	Cyclobarbital	-5.9
14	2-Undecanone, 6,10-dimethyl	-5.9
15	Dodecanoic Acid	-5.8
16	1-Octadecyne	-4.9
17	1,3-Propanediol,2-(hydroxymethyl)-2-nitro-	-4.6
