# Supplementary material for: Integrated Network Pharmacology and Molecular Docking Uncover Multi-Target Actions of Cladophora glomerata–Derived Compounds Against Chronic Obstructive Pulmonary Disease
Source: Int J Mol Sci. 2026 Feb 7;27(4):1619. doi: 10.3390/ijms27041619 (PMC12940951; doi:10.3390/ijms27041619)
Supplement: Supplementary file 1 [file ijms-27-01619-s001.zip › Supplementary Files/Table_S3.pdf]

**Table S3.** Binding Affinity of Cladophora Glomerata derived compounds with target protein TP53

| S.No | Compound Name | Binding Affinity (kcal/mol) |
|---|---|---|
| 1 | 1,2,4-Oxadiazole, 3-(1,3-benzodioxol-5-yl)-5-[4-iodo-1H-pyrazol-1-yl)methyl]- | -6.2 |
| 2 | Quinoline, 1,2,3,4-tetrahydro 1-((2 phenylcyclopropyl)sulfonyl-,trans- | -5.8 |
| 3 | 1H-Pyrrolo[3,4-c]pyridine- 1,3,4(2H,5H)-trione, 6-methyl- | -5.7 |
| 4 | Cyclobarbital | -5 |
| 5 | Phytol | -4.9 |
| 6 | N-Methyl-1-adamantaneacetamide | -4.9 |
| 7 | 3,7,11,15-Tetramethyl-2-hexadecen-1-ol | -4.5 |
| 8 | Bicyclo[3.1.1]heptane, 2,6,6-trimethyl, 2,3-bis-(methylthio) | -4.3 |
| 9 | 1,3-Propanediol,2-(hydroxymethyl)-2-nitro- | -4 |
| 10 | Z,Z,Z-1,4,6,9-Nonadecatetraene | -4 |
| 11 | 9,12- Octadecadienoic acid, methyl ester(E,E)- | -4 |
| 12 | Dodecanoic Acid | -3.8 |
| 13 | 9-Hexadecenoic acid, methyl ester,(Z)- | -3.8 |
| 14 | 2-Undecanone, 6,10-dimethyl | -3.7 |
| 15 | Tetradecanoic acid | -3.6 |
| 16 | Hexadecenoic acid, methyl ester | -3.6 |
| 17 | 1-Octadecyne | -3.5 |
