# Supplementary figures and images for: Integrated Network Pharmacology and Molecular Docking Uncover Multi-Target Actions of Cladophora glomerata–Derived Compounds Against Chronic Obstructive Pulmonary Disease
Source: Int J Mol Sci. 2026 Feb 7;27(4):1619. doi: 10.3390/ijms27041619 (PMC12940951; doi:10.3390/ijms27041619)

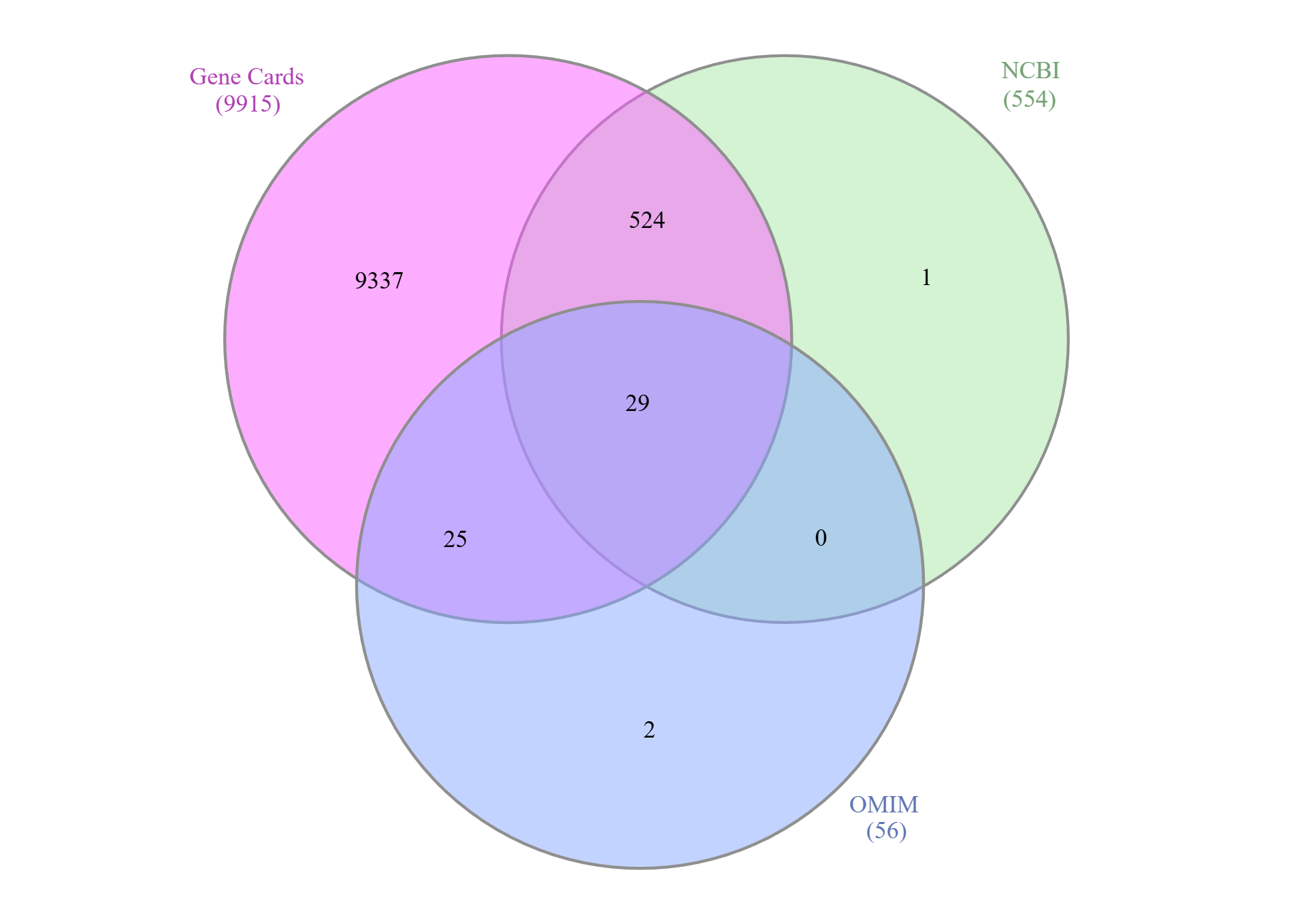

Supplement: Supplementary file 1 [file ijms-27-01619-s001.zip › Figures/figure 1.png]

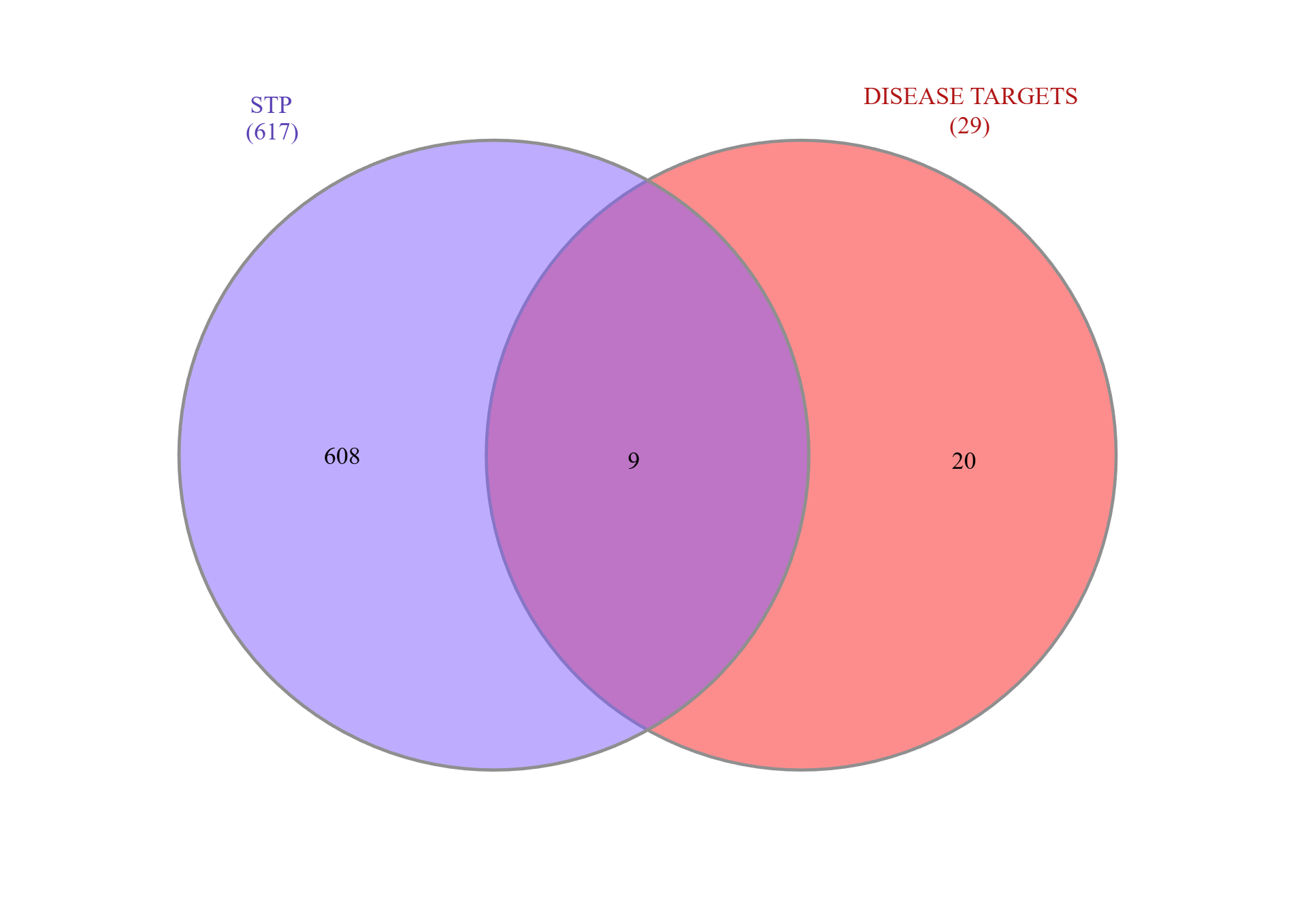

Supplement: Supplementary file 1 [file ijms-27-01619-s001.zip › Figures/figure 2.png]

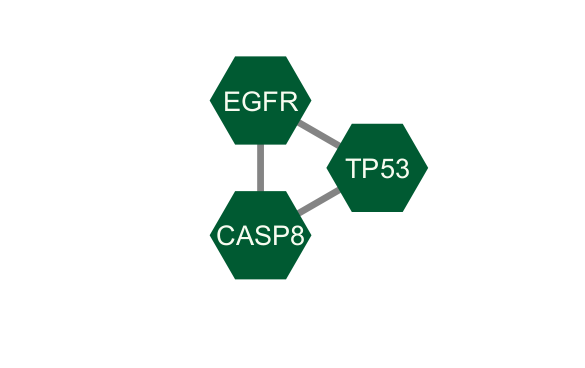

Supplement: Supplementary file 1 [file ijms-27-01619-s001.zip › Figures/figure 3.png]

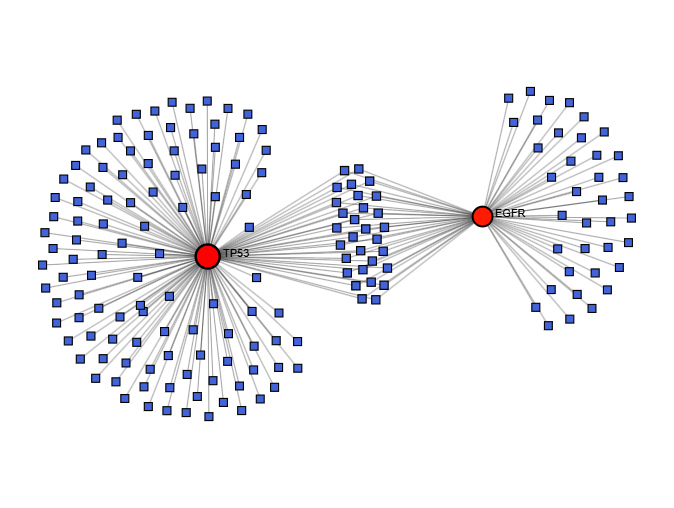

Supplement: Supplementary file 1 [file ijms-27-01619-s001.zip › Figures/figure 4.png]

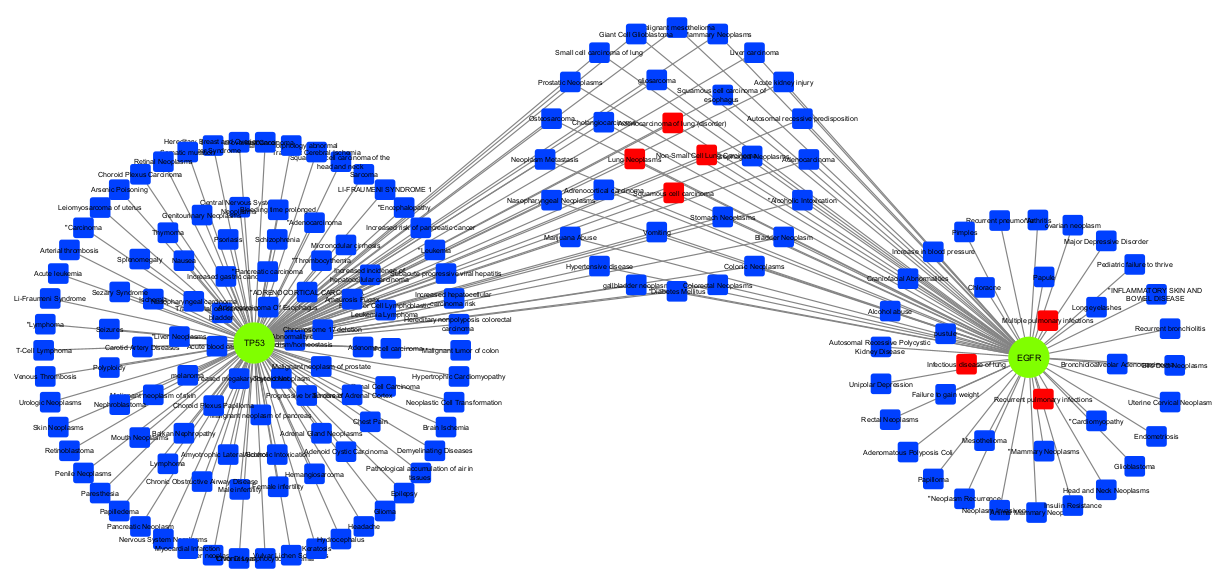

Supplement: Supplementary file 1 [file ijms-27-01619-s001.zip › Figures/figure 5.png]

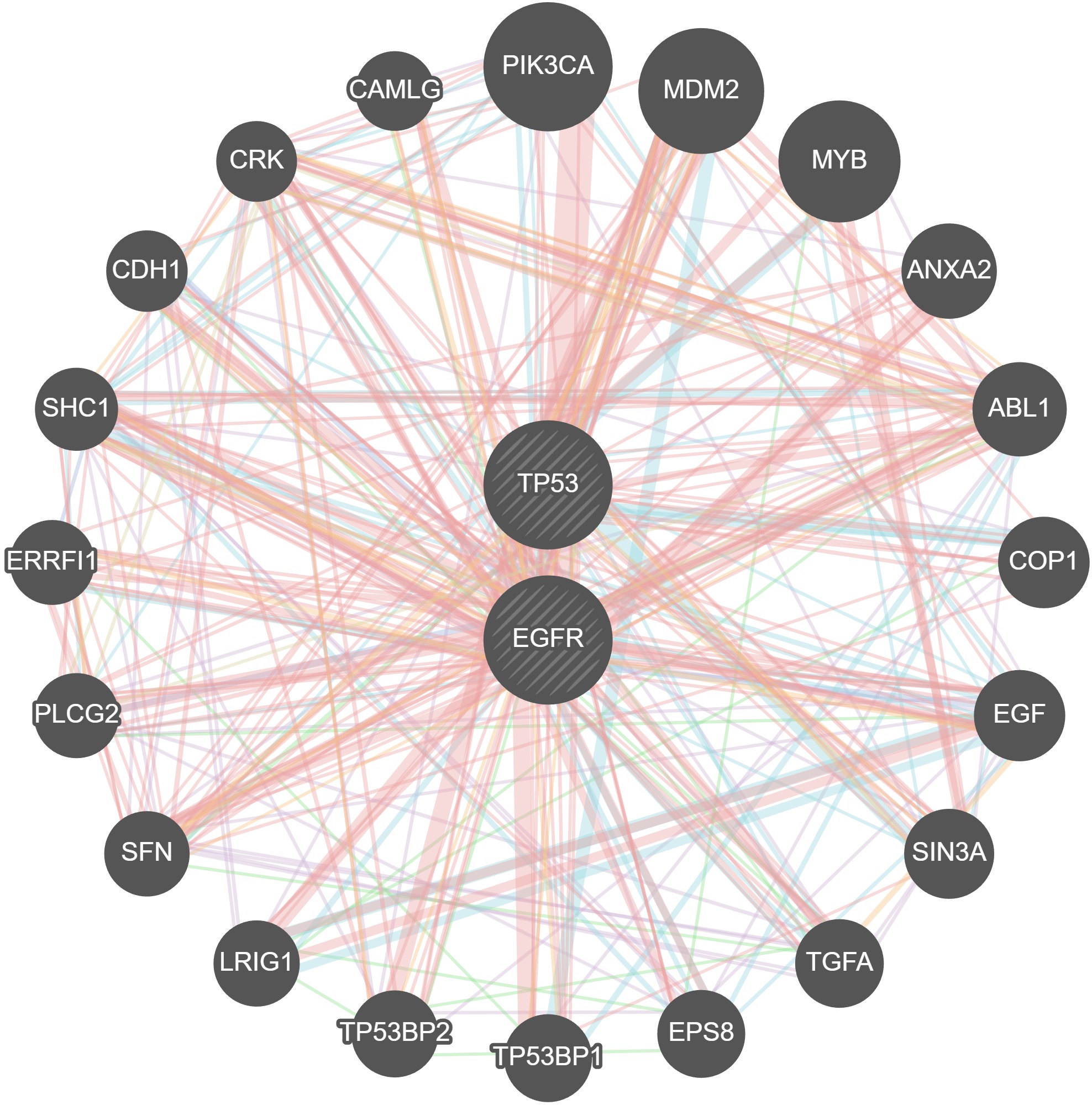

Supplement: Supplementary file 1 [file ijms-27-01619-s001.zip › Figures/figure 6.jpg]

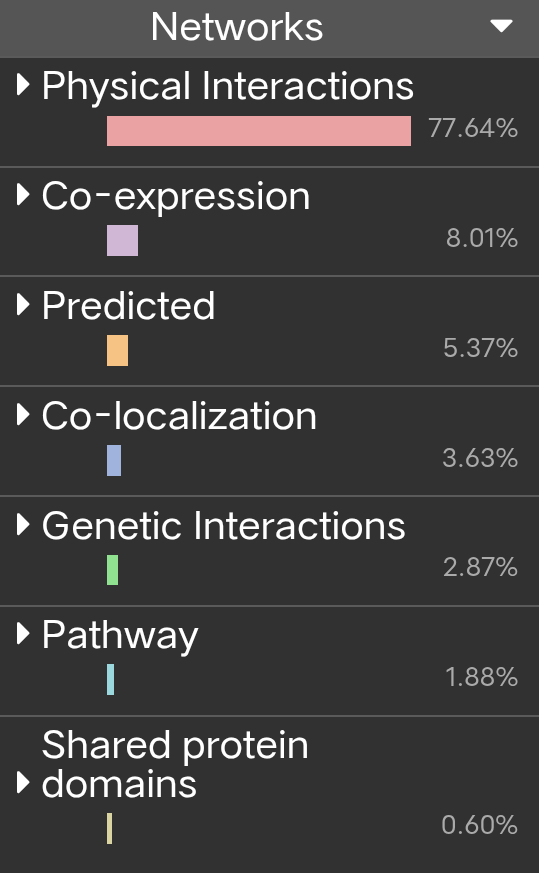

Supplement: Supplementary file 1 [file ijms-27-01619-s001.zip › Figures/figure 6_1.png]

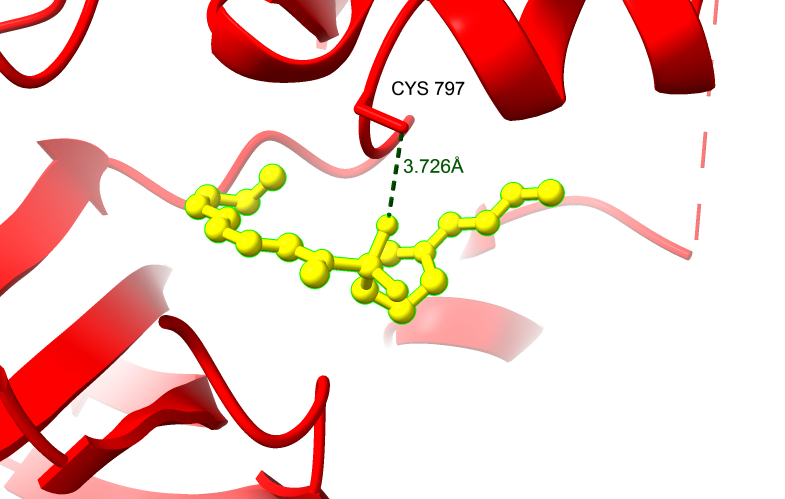

Supplement: Supplementary file 1 [file ijms-27-01619-s001.zip › Figures/Figure 7_1_A.png]

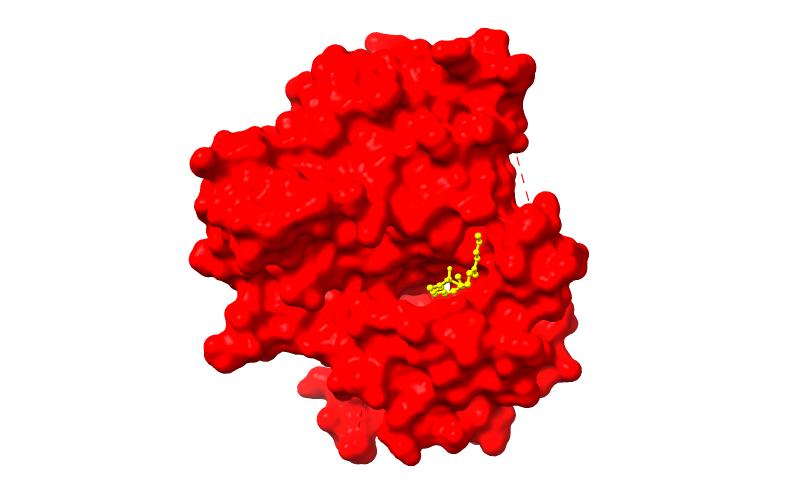

Supplement: Supplementary file 1 [file ijms-27-01619-s001.zip › Figures/Figure 7_1_B.png]

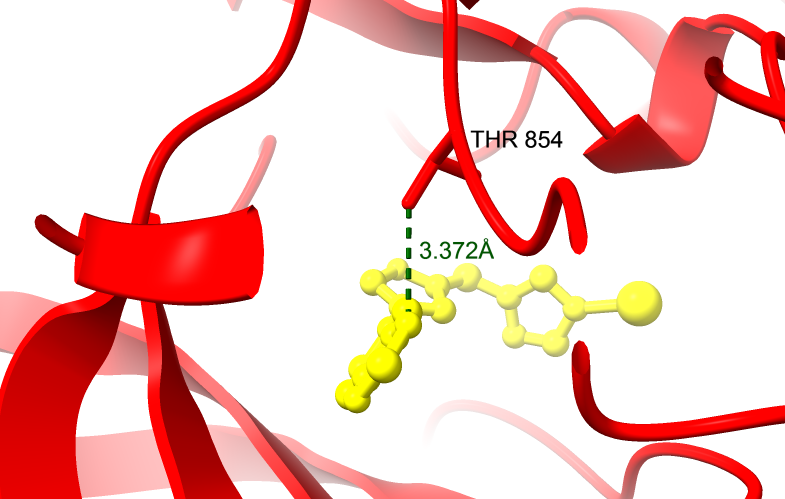

Supplement: Supplementary file 1 [file ijms-27-01619-s001.zip › Figures/Figure 7_2_A.png]

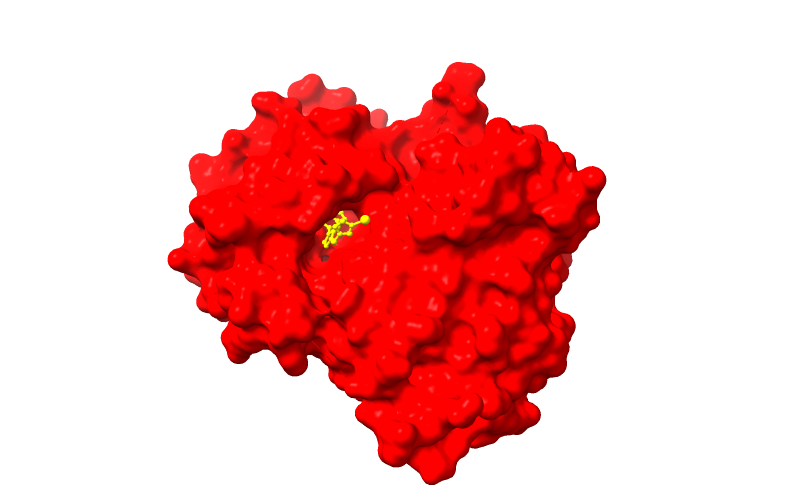

Supplement: Supplementary file 1 [file ijms-27-01619-s001.zip › Figures/Figure 7_2_B.png]

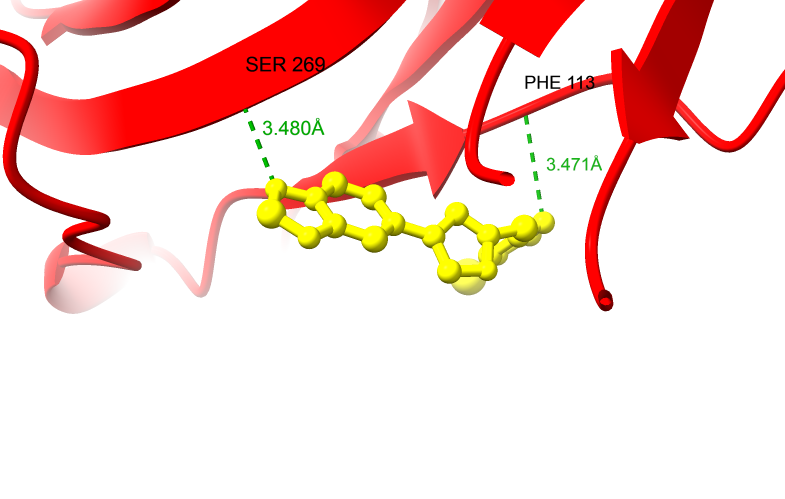

Supplement: Supplementary file 1 [file ijms-27-01619-s001.zip › Figures/Figure 8_1_A.png]

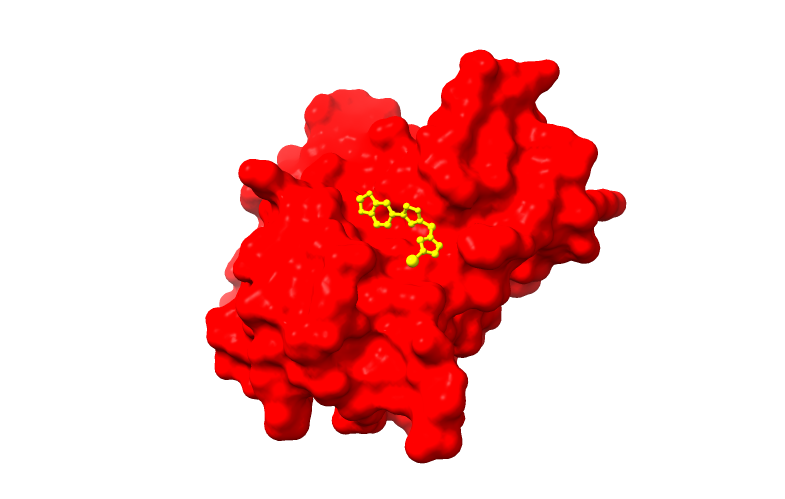

Supplement: Supplementary file 1 [file ijms-27-01619-s001.zip › Figures/Figure 8_1_B.png]

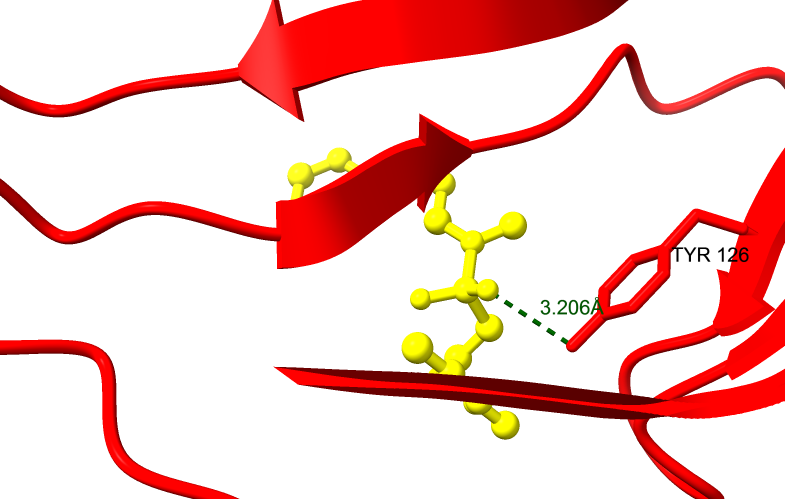

Supplement: Supplementary file 1 [file ijms-27-01619-s001.zip › Figures/Figure 8_2_A.png]

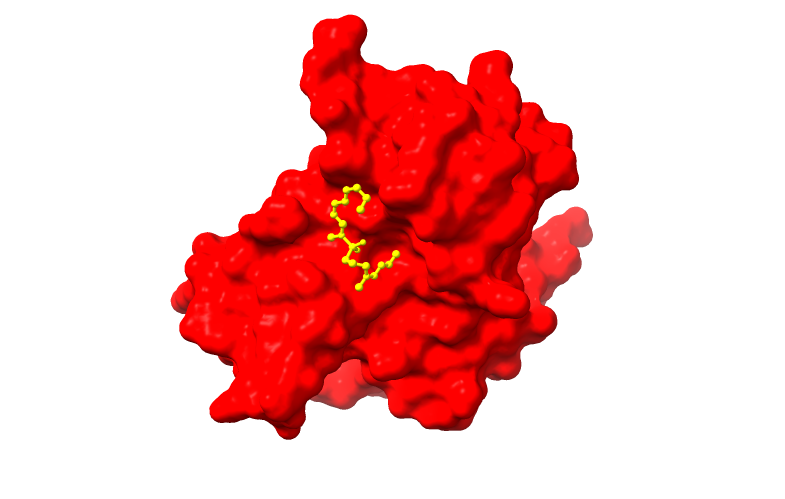

Supplement: Supplementary file 1 [file ijms-27-01619-s001.zip › Figures/Figure 8_2_B.png]
